# Angioedema Coexisting Chronic Spontaneous Urticaria Negatively Influences Patients’ Sense of Coherence, What Results in Susceptibility to Anxiety Symptoms Occurrence

**DOI:** 10.3390/jcm10132852

**Published:** 2021-06-28

**Authors:** Karina Badura-Brzoza, Zenon Brzoza

**Affiliations:** 1 Department of Psychiatry in Tarnowskie Gory, Faculty of Medical Sciences in Zabrze, Medical University of Silesia in Katowice, 42-612 Tarnowskie Gory, Poland; kbbrzoza@sum.edu.pl; 2 Department of Internal Diseases with Division of Allergology, Institute of Medical Sciences, University of Opole, Al. W. Witosa 26, 45-401 Opole, Poland

**Keywords:** sense of coherence, anxiety, urticaria, angioedema

## Abstract

Background: Angioedema coexisting chronic spontaneous urticaria (CSU) is proved to result in patient anxiety occurrence, but the mechanisms and susceptibility patterns are unknown. Sense of coherence (SOC) is one of methods of coping with stress and is defined as a person’s general orientation toward life. We decided to assess SOC disturbances in CSU patients in the context of possible angioedema association. Methods: The study comprised 71 CSU subjects. To analyze disease activity, the Urticaria Activity Score seven-day assessment questionnaire (UAS7) was used. For anxiety assessment, the STAI questionnaire was used. The SOC-29 questionnaire, consisting of questions related to comprehensibility (SOC-C), manageability (SOC-M), and meaningfulness (SOC-Mf), was used to analyze SOC parameters (SOC-T). Results: In patients with coexisting angioedema, we observed statistically significantly lower values of SOC-Mf and SOC-T in comparison to the wheals only group. In the angioedema group, we noticed significant negative correlations between SOC-M and SOC-Mf, as well as SOC-T values and anxiety. In the wheals only group, we proved statistically significant correlations between SOC-Mf and SOC-T and anxiety assessed as a state. Conclusions: It is necessary to identify CSU patients manifesting angioedema as they are more likely to have impaired SOC. Lower SOC in this specific group of patients can be related to anxiety symptoms occurrence and should probably be an indication for psychological support.

## 1. Introduction

In pathophysiology of chronic spontaneous urticaria (CSU), immune, inflammatory, hormonal, and genetic abnormalities should be taken into account [1,2,3]. Symptoms of CSU have a detrimental influence on patients’ quality of life, but researchers’ attention is only incidentally focused on disease-related stress and its effects. Undoubtedly, the symptoms of CSU are the cause of distress for the patients, e.g., itching intensity in CSU was proved to be related to stress [4,5]. We do not know if CSU symptoms, wheals, or angioedema are supposed to cause a similar effect. The proportion of CSU patients manifesting both wheals and angioedema, and those reporting only one type of symptom, is different—a similar number of patients experience both symptoms or wheals only, whereas up to 13% experience angioedema only [6]. Angioedema is proved to result in patients’ anxiety development, but the underlying mechanism and ways of identification of patients who are particularly susceptible to manifest emotional disturbances are not thoroughly explored [7].

Sense of coherence (SOC) is one of the methods of coping with stress. The concept of SOC was introduced by Antonovsky and is defined as a person’s general orientation toward life [8]. SOC is an orientation that enables to choose the best coping strategy when facing a problem. SOC consists of three elements: comprehensibility (conditions the ability to order the received stimulus and facilitates anticipation of events or at least how to understand and order them); manageability (the feeling of possessing the means or resources allowing to affect a situation actively); meaningfulness (conviction that it is worthy to be involved in a given situation, fight, and actively influence one’s life) [8]. In previous studies, SOC was proved to be negatively correlated with stress levels and psychological disturbances [9]. The number of studies concerning values of SOC in allergic and dermatologic diseases is markedly limited. There are very a few data on SOC in CSU, with no study analyzing the potential influence of coexisting angioedema. Our study was designed to assess SOC disturbances in patients suffering from CSU, particularly in the context of their association with angioedema coexistence. We are the first to focus on such novel data, looking for factors indicating susceptibility to emotional disturbance manifestation.

## 2. Material and Methods

Consecutive CSU patients were included into the study. The diagnosis of CSU was established on the basis of medical history and physical examination. The exclusion criteria were other uncontrolled somatic or psychiatric diseases.

To analyze disease activity, the Urticaria Activity Score (UAS) questionnaire was used. This tool calculates its result from the sum of wheals and the intensity of pruritus. A recommended seven-day monitoring period was added up to result in UAS7. For the anxiety assessment, we used the State-Trait Anxiety Inventory (STAI). This tool comprises 20 questions concerning the state anxiety (STAI-S) and 20 questions concerning anxiety assessed as a trait (STAI-T). SOC-29 is a 29-item self-report questionnaire, consisting of questions related to various aspects of life orientation: comprehensibility (SOC-C) (11 questions), manageability (SOC-M) (8 questions), and meaningfulness (SOC-Mf) (10 questions). Answers to questions are placed on a 7-point scale of agreement or disagreement. All items are added up to compute the total value (SOC-T). Obtained results range between 29 and 203. The higher the score, the stronger the sense of coherence. The questionnaire package was accompanied with the information letter and the consent form.

Comparisons between groups were performed by means of the U Mann–Whitney test. Spearman rank order correlation coefficients were computed to explore relations between the examined parameters. P values less than 0.05 were considered statistically significant (Statistica 13.3, Statsoft INC., Tulsa, OK, USA).

The study was conducted according to the guidelines of the Declaration of Helsinki and approved by the Ethics Committee of the Medical University of Silesia, Katowice, Poland (KNW/0022/KB1/18/15). Written informed consent was obtained from all participants.

## 3. Results

In total, 71 CSU subjects (45 females and 26 males, median age: 45 years; range 20–77) were included. Symptoms of recurrent angioedema coexisting wheals (Ae group) were identified in 34 patients (48%) contrary to 37 patients presenting only with wheals (U group). Between these groups, we did not notice any statistically significant differences concerning age or disease duration time (Table 1). In the Ae group, we observed statistically significantly lower values of SOC-Mf and SOC-T in comparison to the U group. We did not notice any other significant differences when comparing other analyzed parameters between our subgroups (Table 1). Statistically significant correlations between analyzed parameters in the Ae group are presented in Table 2. We noticed statistically significant negative correlations between SOC-M and SOC-Mf, as well as SOC-T values and anxiety (Table 2). Additionally, in the U group, we proved a statistically significant correlation between SOC-Mf, as well as SOC-T, and anxiety assessed as a state (Table 3).

## 4. Discussion

In the present study, we aimed to analyze in CSU patients an effect of angioedema symptoms coexisting with wheals on SOC and its potential relation to anxiety symptoms occurrence. We are the first to notice that coexistence of angioedema impairs SOC, particularly in the domains related to meaningfulness, and favors anxiety occurrence. There is a limited number of studies related to SOC and urticaria, and, to the best of our knowledge, no studies have been carried out to examine SOC in relation to angioedema coexistence in CSU patients. We are the first to explore this possible dependency. 

Recent projects proved psychological distress and emotional disturbances as common in patients with CSU [5,10,11,12,13]. According to some data, even 60% of chronic urticaria patients suffer from various psychiatric disturbances [14]. On the other hand, results of previous studies proved that 30–50% of CSU patients manifest angioedema symptoms [15,16,17]. Coexisting angioedema is found to be a risk factor of a more severe urticaria course and symptoms reoccurrence in the 5-year follow-up [15]. In general, an additional effect of angioedema coexisting with wheals on different aspects of the disease course seems to be significant though relatively poorly explored and measured. We know that similar clinical symptoms, such as oedema attacks in the course of hereditary angioedema, can provoke symptoms of depression and anxiety [18]. Clinical experience presents symptoms of angioedema in the course of CSU, especially in a specific location, resulting in emotional disturbances manifestation. 

The detrimental influence of CSU symptoms on patients’ emotional status and quality of life is undoubtable [19]. A strong SOC supports the belief that the world is predictable and underlines an important function of one’s self-agency in influencing the events according to one’s expectations [8]. Patients who present problems with coping manifest susceptibility to emotional disturbance occurrence. Previous studies confirm the relationship between a lower sense of coherence and a higher intensity of depression and anxiety [20]. Patients with chronic asthma and atopic dermatitis presented lower SOC accompanied by higher parameters of depression and anxiety than persons suffering from chronic tinnitus [20]. Another authors after analysis of patients with aspirin-induced asthma and severe chronic asthma conclude that decreased SOC influences the development and severity of depression and anxiety symptoms [21]. Similar dependency of an increased depression and lowered SOC was noticed in patients with atopic dermatitis when compared to persons with remission and persons without allergic diseases [22]. 

There are only a few studies focused on this topic in CSU. We found a study analyzing negative emotions and their relation to the parameters of SOC in the course of chronic urticaria [23]. This study underlines the negative correlation between emotional disturbances and global SOC, as well as its components. Negative dependency between the severity of disease symptoms and total SOC is proved as well [23]. Another study reports lower SOC, higher anxiety, and higher intensity of depression in chronic urticaria subjects when compared to healthy controls [5]. It must be underlined that the above-mentioned studies did not analyze the influence of angioedema on the obtained results. In our study, we noticed that lower SOC in CSU patients with angioedema is related to anxiety symptoms manifestation. The additional advantage and novelty of our study is that, by using the STAI questionnaire, we analyzed different forms of anxiety.

## 5. Conclusions

In our opinion, it is necessary to identify CSU patients with coexisting angioedema as they are more likely to have markedly impaired SOC. Lower SOC in this specific group of patients can be related to anxiety symptoms occurrence and should probably be an indication for psychological support.

## Figures and Tables

**Table 1:** Comparison of analyzed parameters between studied groups of urticaria patients.

	Ae Median (Min–Max)	U Median (Min–Max)	*p*
Age (years)	47.0 (25.0–75.0)	43.5 (20.0–77.0)	ns
Disease duration time (months)	16.5 (3.00–60.0)	24.0 (2.0–51.0)	ns
SOC–C (points)	48.0 (20.0–55.0)	47.0 (35.0–72.0)	ns
SOC-M (points)	42.0 (31.0–57.0)	47.0 (37.0–60.0)	ns
SOC-Mf (points)	36.5 (28.0–44.0)	43.0 (31.0–54.0)	0.02
SOC-T (points)	133.0 (80.0–145.0)	138.0 (106.0–169.0)	0.04
STAI-S (points)	47.0 (27.0–66.0)	44.0 (26.0–67.0)	ns
STAI-T (points)	46.5 (32.0–63.0)	45.0 (29.0–60.0)	ns
UAS7 (points)	18.0 (8.0–30.0)	20.0 (6.0–34.0)	ns

Ae, patients with angioedema coexisting wheals; U, patients with wheals only; SOC-C, sense of coherence-comprehensibility; SOC-M, sense of coherence-manageability; SOC-Mf, sense of coherence-meaningfulness; SOC-T, sense of coherence-total score; STAI-S, state-trait anxiety inventory—state; STAI-T, state-trait anxiety inventory—trait; *p*, statistical significance; ns, statistically nonsignificant.

**Table 2:** Statistically significant correlations between analyzed parameters in urticaria patients manifesting angioedema coexisting wheals.

Parameter	R	*p*
SOC-M & STAI-S	−0.72	0.04
SOC-M & STAI-T	−0.86	0.01
SOC-Mf & STAI-T	−0.76	0.03
SOC-T & STAI-T	−0.86	0.01

SOC-M, sense of coherence-manageability; SOC-Mf, sense of coherence-meaningfulness; SOC-T, sense of coherence-total score; STAI-S, state-trait anxiety inventory—state; STAI-T, state-trait anxiety inventory—trait; R, Spearman coefficient; *p*, statistical significance.

**Table 3:** Statistically significant correlations between analyzed parameters in urticaria patients manifesting wheals only.

Parameter	R	*p*
SOC-Mf & STAI-S	−0.53	0.02
SOC-T & STAI-S	−0.46	0.04

SOC-Mf, sense of coherence-meaningfulness; SOC-T, sense of coherence-total score; STAI-S, state-trait anxiety inventory—state; R, Spearman coefficient; *p*, statistical significance.

## Data Availability

The data presented in this study are available on reasonable request from the corresponding author.
